# In-Flight Transmission of Novel Influenza A (H1N1)

**DOI:** 10.4178/epih/e2010006

**Published:** 2010-05-31

**Authors:** Joon Hyung Kim, Dong-Han Lee, Sang-Sook Shin, Chun Kang, Jin Seok Kim, Byung Yool Jun, Jong-Koo Lee

**Affiliations:** Korea Centers for Disease Control and Prevention, Seoul, Korea.

**Keywords:** Influenza, Human, Transmission, Aircraft, Ventilation, Disease Outbreaks

## Abstract

The Korea Centers for Disease Control and Prevention confirmed two patients, who had taken the same plane from Los Angeles to Seoul, with novel influenza A (H1N1). Through contact tracing, we concluded that the second patient was infected during the flight.

The first H1N1 patient in the Republic of Korea was confirmed by virus isolation at the Korea Centers for Disease Control (KCDC) on May 2, 2009 [1]. The patient was a 52-yr-old nun (patient A) who had travelled through Mexico between April 17 and 25. On April 25, she departed from Mexico and made a transfer in Los Angeles (LA), boarding a Seoul-bound Boeing 747-400. On the morning of her departure, she started to cough and feel febrile. After she arrived in Korea, she isolated herself in her room, fearing that she might have contracted the novel H1N1 virus. On April 27, she reported to a public health center. The next day, she was classified as a probable patient, as test results showed that she was positive for influenza A, but negative for seasonal H1 and H3. As her symptoms were mild, she did not need to be hospitalized. However, the KCDC isolated her in the National Designated Isolation Hospital to prevent the spread of the virus. Later, on May 5, a 44-yr-old woman (patient B) was identified as another H1N1 patient by virus isolation. patient B had driven patient A from the airport to the nunnery, talked with patient A in patient A's room with the door closed for a few minutes, and delivered every meal to patient A until patient A was isolated in the hospital on April 28.

On the day patient A was identified as a probable patient, the KCDC began to distribute oseltamivir to passengers who had sat within 2 meters of the nun and kept them isolated in their houses. There were 338 persons in the craft and 124 were in transit to other countries or had left Korea. Of the 214 people staying in Korea, including 23 crew members, 14 passengers were not contactable and one was the index patient. We followed up on 199 persons, four of whom had symptoms of respiratory illness (Figure 1). By conventional RT-PCR, three of these four patients had a negative result. One of the four patients, a 62-yr-old woman (patient C), was classified as a probable patient on May 3 and was confirmed with H1N1 on May 7. Fever (body temperature 37.9℃), malaise and myalgia developed in patient C on April 29. All of her symptoms were improved by May 2.

Patient C had been staying with her daughter for 6 months in Tucson, Arizona. She started to cough on April 24 just after she had been exposed to an electronic fan all day long. Thus, it was thought to be non-specific. On the morning of April 25, patient C took a flight to LA and then transferred onto the same Seoul-bound flight as patient A.

To determine the origin of her infection, we analyzed patient C's movements. In Tucson, patient C usually spent her time baby-sitting her granddaughter, and made weekly trips to the market. During the two weeks before her flight back to Korea, nobody had visited her house and she had stayed at home, never going out. All other members of the family were healthy. Given that patient C did not go out for two weeks before her initial cough, it is unlikely that she was infected with H1N1 at that time as the incubation period, although unknown, seems to range from 1-7 days, and more likely 1-4 days [2]. Furthermore, since the first confirmed patient in Tucson was reported on May 3 [3], it is highly improbable that patient C was infected with H1N1 in Tucson from asymptomatic household members.

Patient C was driven by her daughter to the Tucson airport. There were few passengers at the Tucson airport and patient C denied talking to anyone there. On the flight to LA, only half of the seats were occupied. Patient C sat next to the window with her daughter beside her. The seats in front of and behind patient C were vacant. No one seemed to be sick on the flight, which took about 90 min. In LA, they had breakfast in an airport restaurant with a few other customers. She does not remember seeing a nun (patient A) during the hour of waiting in front of the gate. It seems unlikely that patient C was infected with H1N1 during the domestic flight or at the airports.

Thus, given the low probability of patient C being infected before she boarded the Seoul-bound plane and because she was in the vicinity of the nun during the infectious period (from one day before to seven days following the onset of illness [2]), it appears highly likely that patient C contracted the H1N1 virus from the nun. The seat number of patient A (the nun) was 29J and that of patient C was 35H. As the distance between each seat is 0.83 meters, and taking into account that there is an empty space between rows 31 and 32, the gap between the two patients' seats was more than 5 meters. Passengers near the nun stated that the nun coughed vigorously, but patient C denied hearing any coughing or seeing the nun before, during or after the flight. The flight lasted approximately 13 hr, and both patients denied moving around except to use the restrooms. Patient A used the restroom three times while patient C went eight times. Both of them used the same adjacent four lavatories, but neither remembered exactly when or which ones they used.

Since patient C never saw the nun, it is unlikely that she became infected through droplet transmission by close contact. However if patient C used the same lavatory as the nun, she might have had direct contact with the contaminated environment such as faucets, walls, or knobs. Another possible transmission route is that she might have inhaled the droplets left in the air of the lavatory. Usually aircrafts are flown at an altitude of 30,000 to 36,000 feet, at which 3 µm particles fall at a speed of 1.4 cm/sec [4]. Patient C might have entered the same lavatory during the falling of droplets. Though patient A and C did not exactly remember which lavatories they used, they used some of the four lavatories near their seats.

Another route to consider is via airborne transmission. In the modern aircraft, the air in the cabin is provided by an environmental control system which is designed to control cabin pressure and temperature, maintain the quality of the air, and filter/dissipate any particulate matter, smoke or odors [5]. As a result, in the cabin, air flows vertically downwards from the ceiling to the floor [6]. In addition, normal air exchange rates in airline cabins range from 15 to 20 air changes per hour [7]. When passengers expel potential respiratory pathogens, the infectious agents usually enter laminar flow and are diluted by frequent air exchanges [6]. However, although aircraft ventilation systems are well equipped, some conditions, such as movements of passengers or staff, or shaking of the aircraft, may disrupt the laminar air flow in the cabin and cause turbulence, and instances of advective flow moving back toward the rear of the cabin have been reported [8]. Moreover, the very low relative air humidity in the cabin (mean 6%, range 1-27%) [9] makes the influenza virus more transmissible [10]. As there are multiple reports of in-flight transmission [11-13], it is possible that the virus could have escaped the ventilation system and reached the other passengers.

One report calculated the potential for the within-flight transmission of influenza A (H1N1) [14]. According to the report, if the source case travels in Economy class, five to ten infections could occur during an 11-hr flight. In our study, we found only one patient who seemed to have been infected during the flight. The difference between the expected and actual transmission might be attributable to passengers who were not available for interview and the airflow in the cabin. There might have been another influenza A (H1N1) patient among the uninterviewed patients. Moreover, the calculated transmission rate assumed that the air in each cabin is well mixed [14], but in reality the air is not freely mixed because air flows vertically downwards despite some turbulence.

The risk of the in-flight transmission of H1N1 is determined by multiple factors: the infectivity of the source, the virulence of the virus, the size of the cabin, the ventilation rate, the humidity and temperature, host-specific factors, and the duration of the flight. A properly working ventilation system keeps the possibility of airborne transmission relatively low. However, if a H1N1-infected patient is on board, passengers and crew members stand a good chance of direct or even indirect exposure to the H1N1 virus, especially during long flights. Though the report [14] suggesting possible in-flight transmission was published before, our report is the first report defining the in-flight transmission of novel influenza A (H1N1).

Therefore, during the early stages of a pandemic, it would be helpful to check the health status of passengers before boarding. Passengers suspected of being infected with H1N1 should not be allowed to board the aircraft, thus slowing down the worldwide spread of the virus. If a suspected patient is found on board, the patient should be moved toward the back of the aircraft, emptying seats around the suspected patient, and designating one lavatory for only the patient's use. If this is not possible, the health authorities may need to consider giving all passengers, not only those sitting near the patient, anti-viral medication. In conclusion, health authorities should not disregard the possibility of in-flight transmission to passengers sitting more than 2 meters from a patient, and should take necessary precautions to prevent the global spread of novel influenza A (H1N1) virus.

## Figures and Tables

**Figure 1:**
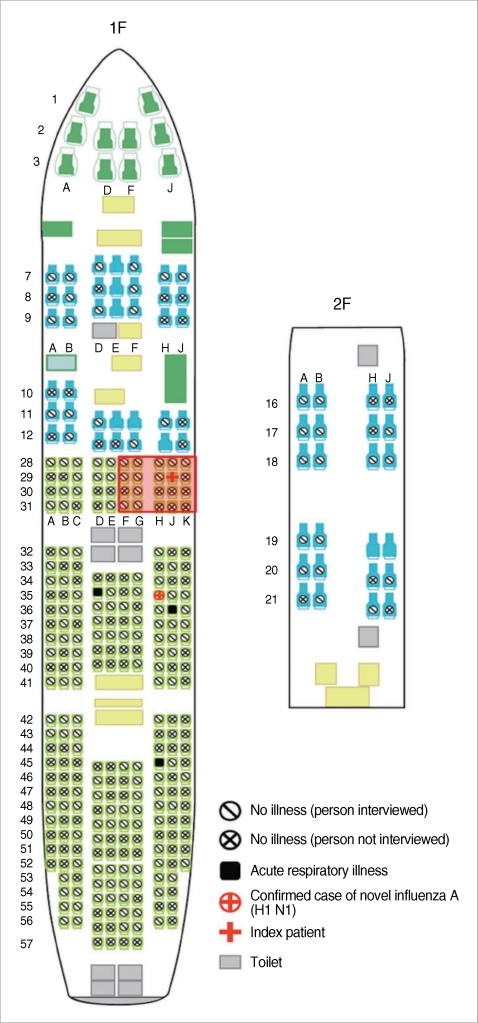
Schematic diagram of the Boeing 747-400 Aircraft from LA to Seoul. Four people had symptoms of respiratory illness. One who sat in seat 35D complained of a stuffy nose and a slightly sore throat. Another who sat in seat 36J had just a sore throat and an intermittent cough, which started on April 27. The other who sat in seat 45H felt feverish and had rhinorrhea, which started before boarding. The passengers who were interviewed and sat in the red rectangle received oseltamivir for prophylaxis.
